# Primary school-based food environment intervention increases diet diversity: Project Daire, a cluster randomized controlled trial

**DOI:** 10.1186/s12966-025-01842-4

**Published:** 2025-11-21

**Authors:** Dilara Olgacher, Ciara Wallace, Sarah F. Brennan, Fiona Lavelle, Sarah E. Moore, Michelle C. McKinley, Patrick McCole, Ruth F. Hunter, Laura Dunne, Chris R. Cardwell, Danielle McCarthy, Jayne V. Woodside

**Affiliations:** 1 Centre for Public Health, Queen’s University Belfast, Belfast, UK; 2 Institute for Global Food Security, Queen’s University Belfast, Belfast, UK; 3 Department of Nutritional Sciences, School of Life Course and Population Sciences, King’s College London, London, UK; 4 School of Business, Maynooth University, Maynooth, County Kildare Ireland; 5 School of Social Sciences, Education and Social Work, Queen’s University Belfast, Belfast, UK; 6 Nutrition Talent Limited, Bangor, UK

**Keywords:** School-based intervention, Children, Diet diversity, Diet quality, Whole-school approach, Food environment, Food education

## Abstract

**Background:**

This study explored the effects of Project Daire, a school-based food intervention, on secondary dietary outcomes Diet Diversity Score (DDS) and Diet Quality Score (DQS), among 6–7 and 10-11-year-old children.

**Methods:**

A randomised-controlled, factorial design trial was conducted in 15 Northern Ireland primary schools across four intervention arms: Nourish, Engage, Nourish and Engage, and Control (Delayed). Nourish modified the school food environment and increased exposure to local foods, while Engage delivered educational activities on nutrition, food, and agriculture. Food consumption data were collected at baseline and at up to a 6-month follow-up. DDS and DQS (at home, at school and/or total) were determined based on the UK Eatwell Guide.

**Results:**

A total of 445 children aged 6–7 and 458 aged 10–11 completed the trial. Among the 10-11-year-olds who received the Nourish intervention, significant increases were observed in the school DDS (adjusted mean difference = 2.79, 95% CI: 1.44–4.14; *p* < 0.001) and total DDS (adjusted mean difference = 1.55, 95% CI: 0.65–2.44; *p* = 0.001) compared to their counterparts who did not receive it. No such changes were observed in the DDS of 6-7-year-olds in the Nourish group, nor in either age group receiving the Engage intervention. The DQS of both age groups remained unchanged across all intervention groups.

**Conclusions:**

The Nourish intervention was associated with improved dietary diversity among older children through modifications to the whole-school environment. However, the absence of measurable effects on diet quality highlights the need for future iterations of Project Daire to incorporate additional strategies. These should include targeted approaches to improve diet quality, foster active parental engagement, utilize validated dietary assessment tools, and ensure sustained implementation.

**Trial registration:**

ClinicalTrials.gov Identifier: NCT04277312.

**Supplementary Information:**

The online version contains supplementary material available at 10.1186/s12966-025-01842-4.

## Background

Despite ongoing public health efforts, children’s diets in the United Kingdom (UK) have not improved as intended over recent decades. The National Diet and Nutrition Survey reported that fewer than 15% of school-aged children met the government’s “5-a-day” fruit and vegetable (FV) recommendation, while average intakes of free sugars and saturated fats remained above recommended thresholds [1]. These concerns are likely compounded by widening dietary inequalities and broader socioeconomic pressures, including the cost-of-living crisis, which constrain families’ ability to access affordable diets aligned with national guidance [2, 3]. Children from lower-income households remain at greater risk, experiencing poorer diet quality than their peers from more advantaged households [1, 4]. The quality of children’s diets not only influences their immediate health and well-being but is also likely to persist into adulthood [5], influencing future risk of non-communicable diseases, such as type 2 diabetes and cardiovascular diseases [6–8]. Addressing these challenges through effective and sustainable interventions therefore remains a critical public health priority in the UK [9].

Schools provide an important setting for food and nutrition interventions, given their reach across large and diverse populations of children over extended periods [8–10]. Many earlier school-based interventions often targeted consumption of single nutrients or food categories, such as FV and sugar-sweetened beverages. Although some positive effects were observed, findings were inconsistent [6–8], reflecting the complexity of dietary behaviours [11]. Foods are not consumed in isolation but as part of an overall diet, where interactions among nutrients and food components collectively influence health outcomes. Consequently, a whole-diet approach offers greater potential to shape dietary habits and ensure nutritional adequacy [11–13]. The World Health Organization’s Health Promoting School framework advocates for a ‘whole-school’ approach, which integrates strategies across the curriculum, school social and physical environments, and family and community engagement, to promote children’s health and well-being [14]. Multi-component interventions of this type can broaden children’s exposure to diverse foods, embed nutrition education, and encourage positive dietary changes [8, 11]. Such comprehensive strategies have the potential to improve overall diet quality and related health outcomes. However, to realize this potential, the development and rigorous evaluation of school-based interventions with high-quality study designs remain essential.

In this context, Project Daire [15] explored the effects of two school-based food interventions, ‘Nourish’ and ‘Engage’, on health-related quality of life, wellbeing, and dietary behaviours among primary school children in socioeconomically disadvantaged areas of Northern Ireland. Nourish, which focused on modifying the whole-school food environment, was associated with a range of positive outcomes, including improvements in children’s emotional and behavioural well-being, food consumption, cooking competence, knowledge of seasonal vegetables, willingness to try new foods, and understanding of food labels. In contrast, Engage, which delivered food education, did not demonstrate the similar level of effectiveness. Subgroup analysis indicated that boys who participated in Nourish were more likely to experience improvements in health-related quality of life and emotional and behavioural well-being than their peers who did not receive this intervention. These findings highlight the value of examining subgroup responses, as some groups, such as boys, may benefit more than others. Insights of this kind can inform the refinement of future iterations of Project Daire to ensure that interventions are responsive to the needs of all children.

The initial evaluation of Project Daire focused on changes in the consumption of individual food items. While these findings provided valuable insights, such an approach does not fully reflect the complexity of children’s diets or capture the broader impact of the interventions. To build on this work, a more comprehensive assessment of dietary intake is warranted [16–18]. The present study therefore extends the earlier analysis by investigating the effects of Nourish and Engage on overall diet diversity and quality, while also examining potential gender-based differences. For this purpose, diet diversity and quality scores, widely recognised as proxy indicators of nutritional adequacy and overall diet healthfulness in children [16, 17, 19, 20], were developed and applied to the Project Daire dietary intake data.

## Methods

### Study design

Daire was a factorial design cluster randomized controlled trial designed to run over a six-month period (January/February to May/June 2019). In practice, the duration of intervention delivery varied between 2.5 and 5 months across schools, as some commenced the programme later than scheduled due to logistical challenges, particularly the demands of busy school timetables [15]. Follow-up data collection was conducted six months after the originally scheduled baseline, in line with the study design. Primary schools from areas with varying levels of socioeconomic disadvantage across Northern Ireland were randomized to one of four intervention arms: (i) Nourish, (ii) Engage, (iii) Nourish and Engage and (iv) Control (Delayed). Socioeconomic disadvantage was determined using the Northern Ireland Multiple Deprivation Measure 2017, based on school postcode. The study was approved by the School of Social Sciences, Education and Social Work Ethics Committee, Queen’s University Belfast (Reference number 038_1819). The trial adhered to the Consolidated Standards of Reporting Trials (CONSORT) 2010 guidelines (consort-spirit.org) and was registered with the National Institute of Health (NIH) U.S. National Library of Medicine Clinical Trials.gov (ID: NCT04277312). The CONSORT checklist for cluster trials is provided in Additional File 1. A detailed description of Project Daire and main outcomes has been published elsewhere [15].

### Recruitment

All mainstream primary schools in the North West region of Northern Ireland were invited to participate in the study if they agreed to (i) be randomly allocated to an intervention, (ii) implement the intervention with their students and (iii) facilitate data collection in their setting. Students were eligible to participate if they were enrolled in the 6–7 or 10–11-year-old class groups during the academic year from September 2018 to December 2018. These age groups were selected based on teacher recommendations to avoid classes involved in formal assessments within the Northern Ireland primary school system. The inclusion of both younger and older primary schoolchildren also enabled consideration of potential differences in dietary autonomy and caregiver influence. Parents received an information sheet about the purpose and aims of the study, the randomization procedure and the nature of the interventions. Parents who did not wish their child to participate in data collection were requested to complete and return an opt-out consent form. Children whose parents submitted this form were excluded from all data collection activities.

### Interventions

The development of the ‘Nourish’ and ‘Engage’ interventions for 6–7 and 10–11-year-old children was guided through collaboration between the study team and school food stakeholders, including school principals, teachers, caterers and local food producers. A detailed description of the intervention components is provided in the TIDieR Checklist **(Additional File 2)**. The Nourish intervention aimed to modify the school food environment to promote children’s interest in food and understanding of food groups. It included food-related experiences, such as food preparation and cooking, along with exposure to local products to increase their interest in food. The intervention was supported by provision of healthy snacks, resources to improve school food presentation, cookery equipment, recipes, sensory education materials, catering for school events, tasting days and recommendations to optimise implementation of school food policies. Engage was an age-appropriate, cross-curricular educational intervention compromised of interactive lessons on food, agriculture and nutrition. The lessons were organised into three main topics: ‘Food Futures’, ‘Farm to Fork’, and ‘Pleasure on a Plate’ and were mainly delivered by teachers or, in some instances, by guest speakers. Schools were also offered opportunities to visit local food industry partners, including a bakery, mushroom farm, poultry producer, and fishery. These visits were designed to provide children with direct exposure to food production and processing practices, helping to build their understanding of food origins and local food systems. Teachers were instructed about the intervention and provided with materials (e.g., videos, books, worksheets, games) and strategies to integrate key concepts into existing curricula and facilitate the classroom sessions. Schools allocated to ‘Nourish and Engage’ received both the Nourish and Engage interventions combined. Control schools were offered all Engage intervention materials for use in the following academic year and £500 for school funds at the end of final data collection. Schools were randomly allocated to ‘Nourish’, ‘Engage’, ‘Nourish and Engage’ or Control (Delayed) after recruitment and baseline data collection, using STATA-generated block sizes of *n* = 4. Detailed intervention components are provided in Additional file 3.

### Data collection

Data on foods consumed were collected using age-appropriate questionnaires administered at baseline and follow-up at 6 months. The questionnaire, developed by the study team, was based on the validated Child and Diet Evaluation Tool (CADET) [21] and modified to include a table-format food list with accompanying pictures to capture foods ‘ever’ consumed. For 6–7-year-old children, a condensed 29-item food list was administered to record overall consumption. For 10–11-year-old children, two 52-item food lists were provided, divided into seven categories (Fruits, Vegetables, Starchy Carbohydrates, Dairy, Meat/Fish/Eggs/Pulses, Snacks, Drinks) to record food consumed at home and school separately **(Additional File 4)**. Response options included: ‘Yes! I sometimes eat or drink this’, ‘No! I never eat or drink this’, or ‘I’m not sure if I eat or drink this’. The questionnaires were self-completed by the children, with assistance from teachers and researchers as needed. Parental or guardian assistance with the home section of the questionnaire was recommended. Although researchers were aware of the intervention due to the nature of data collection and delivery, the researcher who performed data analysis remained blinded to the intervention allocation.

### Development of diet diversity score (DDS) and diet quality score (DQS)

We adopted an approach similar to that of Scheelbeek et al. [22] to develop the Diet Diversity Score (DDS) and Diet Quality Score (DQS) using questionnaire data based on the Eatwell Guide [23], the UK’s food-based dietary guidelines. Responses of ‘no’ and ‘not sure’ were recoded as ‘no’ and compared with ‘yes’ to reflect adherence to the Eatwell Guide. Food items were categorized into core food groups (i.e., FV, carbohydrates, dairy and protein) or discretionary foods high in fat, sugar, and salt (HFSS), based on their alignment with these guidelines. The DDS was designed to measure the overall variety of food items consumed within core food groups rather than classify foods dichotomously as ‘healthy’ or ‘less healthy’. Core food items were scored + 1 if consumed and 0 if not. Discretionary HFSS foods, which do not contribute to dietary diversity within core food groups, were excluded from the diversity measure and scored 0. The DQS was designed to assess the overall quality of the diet by considering both dietary variety and alignment with the Eatwell Guide. Core food items were scored as in the DDS (+ 1 if consumed, 0 if not). However, core food items less aligned with the Eatwell Guide recommendations, such as pancakes, scones, fruit bread, or chicken nuggets/burgers, were penalized within the DQS scoring. These items, although part of a core food group, were scored − 1 if consumed due to their higher fat, sugar, or salt content. Discretionary HFSS foods were also scored − 1 if consumed and + 1 if not. This approach incentivizes the limitation of foods recommended for less frequent consumption while encouraging the intake of nutrient-dense foods within core food groups, thereby better reflecting adherence to the Eatwell Guide. Table 1 outlines the scoring of food items for **(a)** 6–7-year-old and **(b)** 10–11-year-old children based on their reported food consumption. Scores for each questionnaire item were summed to obtain overall DDS and DQS. Higher DDS scores reflect a greater dietary variety across core food groups, while higher DQS scores indicate greater adherence to Eatwell Guide by balancing consumption of core food groups and limiting intake of discretionary HFSS foods. For 6–7-year-old children, a single DDS and DQS were calculated based on their overall food consumption, as recorded in the 29-item food list. In contrast, for 10–11-year-old children, DDS and DQS were calculated separately for foods consumed at school and at home, referred to as school DDS/DQS and home DDS/DQS, respectively. To capture overall dietary patterns for this age group, combined scores from both home and school were calculated and are referred to as total DDS and total DQS.

**Table 1:** Diet diversity score and diet quality score developed from FFQ data for (a) 6–7 and (b) 10–11 years old children. **(a)** 6–7 years old children

**Food Item**		**DDS**		**DQS**	
		**Yes**	**No**	**Yes**	**No**
**(a)** 6-7 years old children					
Fruit	Fruit and Vegetables	+1	0	+1	0
Vegetables	Fruit and Vegetables	+1	0	+1	0
Potatoes, mash, jacket potato, chips, waffles, potato faces	Carbohydrates	+1	0	+1	0
Rice/Pasta	Carbohydrates	+1	0	+1	0
Porridge/ReadyBrek/ Weetabix	Carbohydrates	+1	0	+1	0
Other breakfast cereals	Carbohydrates	+1	0	+1	0
Pancakes/ scones/ fruit bread^a^	Carbohydrates	+1	0	-1	+1
Milk to drink/on cereal	Dairy	+1	0	+1	0
Cheese	Dairy	+1	0	+1	0
Yogurts	Dairy	+1	0	+1	0
Ice cream/milky pudding e.g., custard	Foods high in fat, salt and sugar	0	0	-1	+1
Chicken sliced (no sauce)	Protein	+1	0	+1	0
Chicken nuggets/burgers^a^	Protein	+1	0	-1	+1
Beef	Protein	+1	0	+1	0
Bacon/ham/sausages^a^	Protein	+1	0	-1	+1
Lamb	Protein	+1	0	+1	0
Eggs	Protein	+1	0	+1	0
Fish fillet/tuna	Protein	+1	0	+1	0
Fishfingers/fish in batter	Protein	+1	0	+1	0
Nuts	Protein	+1	0	+1	0
Crisps	Foods high in fat, salt and sugar	0	0	-1	+1
Biscuits/chocolate	Foods high in fat, salt and sugar	0	0	-1	+1
Sweets	Foods high in fat, salt and sugar	0	0	-1	+1
Cakes/buns/muffins	Foods high in fat, salt and sugar	0	0	-1	+1
Fizzy Drink	Foods high in fat, salt and sugar	0	0	-1	+1
Milkshakes	Foods high in fat, salt and sugar	0	0	-1	+1
Water	N/A	0	0	0	0
Juice	N/A	0	0	0	0
**(b)** 10-11 years old children					
Apples	Fruit and Vegetables	+1	0	+1	0
Grapes	Fruit and Vegetables	+1	0	+1	0
Bananas	Fruit and Vegetables	+1	0	+1	0
Strawberries	Fruit and Vegetables	+1	0	+1	0
Pineapple	Fruit and Vegetables	+1	0	+1	0
Mushrooms	Fruit and Vegetables	+1	0	+1	0
Broccoli	Fruit and Vegetables	+1	0	+1	0
Carrots	Fruit and Vegetables	+1	0	+1	0
Peas/Sweetcorn	Fruit and Vegetables	+1	0	+1	0
Stir-fried Vegetables	Fruit and Vegetables	+1	0	+1	0
Salad (tomatoes, leaves or cucumber)	Fruit and Vegetables	+1	0	+1	0
Baked Beans	Protein	+1	0	+1	0
Potatoes	Carbohydrates	+1	0	+1	0
Chips/Potato Faces/Waffles^a^	Carbohydrates	+1	0	-1	+1
Rice/Pasta	Carbohydrates	+1	0	+1	0
White bread^a^	Carbohydrates	+1	0	-1	+1
Brown/wholemeal bread	Carbohydrates	+1	0	+1	0
Porridge/ReadyBrek	Carbohydrates	+1	0	+1	0
Cornflakes/Rice Krispies	Carbohydrates	+1	0	+1	0
Frosties/Coco-pops^a^	Carbohydrates	+1	0	-1	+1
Weetabix/ Bran flakes	Carbohydrates	+1	0	+1	0
Pancakes/scones/ fruit bread^a^	Carbohydrates	+1	0	-1	+1
Crackers/ breadsticks	Carbohydrates	+1	0	+1	0
Milk to drink	Dairy	+1	0	+1	0
Milk on cereal	Dairy	+1	0	+1	0
Cheddar cheese	Dairy	+1	0	+1	0
Cheese spread	Dairy	0	0	-1	+1
Margarine/ butter on toast/ sandwiches	N/A	0	0	0	0
Yogurts	Dairy	+1	0	+1	0
Chicken sliced (no sauce)	Protein	+1	0	+1	0
Chicken nuggets/burgers^a^	Protein	+1	0	-1	+1
Beef	Protein	+1	0	+1	0
Bacon/ham^a^	Protein	+1	0	-1	+1
Sausages	Protein	0	0	-1	+1
Lamb	Protein	+1	0	+1	0
Meat in pastry	Protein	0	0	-1	+1
Eggs	Protein	+1	0	+1	0
Fish fillet	Protein	+1	0	+1	0
Tuna	Protein	+1	0	+1	0
Fish fingers/fish in batter	Protein	0	0	-1	+1
Nuts	Protein	+1	0	+1	0
Ice cream/milky pudding e.g., custard	Foods high in fat, salt and sugar	0	0	-1	+1
Crisps	Foods high in fat, salt and sugar	0	0	-1	+1
Biscuits	Foods high in fat, salt and sugar	0	0	-1	+1
Sweets	Foods high in fat, salt and sugar	0	0	-1	+1
Chocolate	Foods high in fat, salt and sugar	0	0	-1	+1
Cakes/buns/muffins	Foods high in fat, salt and sugar	0	0	-1	+1
Fizzy drink	Foods high in fat, salt and sugar	0	0	-1	+1
Milkshakes	Foods high in fat, salt and sugar	0	0	-1	+1
Water	N/A	0	0	0	0
Juice	N/A	0	0	0	0
Tea/Coffee	N/A	0	0	0	0

^a^Foods items from core food groups that tend to be high in fat, sugar or salt and are therefore less aligned with the recommendations of Eatwell Guide

### Sample size and power

A sample size calculation was conducted for the primary outcomes of Project Daire: emotional and behavioural difficulties, measured with the Strengths and Difficulties Questionnaire (SDQ), and health-related quality of life, measured with KIDSCREEN-10. The factorial design compared Nourish versus No Nourish and Engage versus No Engage, although the study was not powered to assess interactions between the two interventions. Calculations assumed that 12 schools (six intervention and six control) would complete the study, with up to 40 pupils per target year group (Primary 3 or Primary 7) in each school. Based on a standard deviation (SD) of 4.5 for the SDQ [24] and an intracluster correlation coefficient (ICC) of 0.10 informed by prior analyses [25, 26], the study had > 80% power at the 5% significance level to detect a 2.8-point difference in SDQ scores. For KIDSCREEN-10, assuming an SD of 9.0 and the same ICC, the detectable difference was 5.6 points. This required a total sample of 12 schools with approximately 80 pupils each (40 aged 6–7 years and 40 aged 10–11 years), giving 960 children. To account for potential attrition and smaller class sizes, the recruitment target was increased by 20% to 1,152 pupils. These calculations were undertaken for the whole sample, as the primary outcomes were analysed by combining both age groups. In contrast, the secondary dietary outcomes (DDS, DQS) were derived from age-specific dietary questionnaires and therefore analysed separately by age group. No power calculations were performed for dietary outcomes, subgroup comparisons, or interaction effects, which were therefore designated exploratory.

### Statistical analysis

A random intercept multi-level model, with children nested within schools, was used to assess the effects of each intervention (‘Nourish’ and ‘Engage’) on DDS and DQS, accounting for baseline scores and clustering at the school level. Given the factorial trial design, ‘Nourish’ and ‘Engage’, along with baseline scores, were included as independent variables, with the endpoint score as the dependent variable [27]. Mean differences, along with 95% confidence intervals (95% CIs), were calculated for the ‘Nourish’ and ‘Engage’ groups compared to the No Nourish and No Engage groups, adjusted for baseline scores. DDS and DQS were also analysed by gender, followed by an interaction test to examine whether the intervention effects differed between boys and girls. All *P* values were two-tailed, with statistical significance level set at *P* < 0.05. Analyses were conducted using STATA (Version 18, StataCorp LLC, College Station, TX).

## Results

### Baseline characteristics

A CONSORT flow diagram presenting the progression of clusters through the study has been published elsewhere [15]. Fifteen primary schools were randomized for the study, and a total of 903 children (*n* = 445 aged 6–7 years; and *n* = 458 aged 10–11 years) were enrolled [15]. Table 2 provides a summary of the baseline characteristics of participating schools and children.

**Table 2:** Baseline characteristics of schools and children by intervention arms

	Nourish	No Nourish	Engage	No Engage
Number of schools	8	7	7	8
Location				
Urban	4	1	1	4
Rural	4	6	6	4
Age				
6–7 years	320 (54.1)	230 (50.7)	277 (47.8)	273 (48.3)
10–11 years	272 (45.9)	237 (50.5)	254 (47.6)	255 (53.0)
Sex				
Male	297 (50.2)	236 (50.5)	253 (47.6)	280 (53.0)
Female	295 (49.8)	231 (49.5)	278 (52.4)	248 (47.0)

Data were quoted as means (standard deviations) for continuous variables or as numbers (percentages) for categorical variables

### Diet diversity score and diet quality score

Age-specific results for the effects of the Nourish and Engage interventions on DDS and DQS are shown in Table 3. Among children aged 10–11 years, those receiving the Nourish intervention demonstrated an increase in school DDS, from 15.8 at baseline to 17.5 at endpoint, while a decrease was observed in those not receiving Nourish (14.2 to 13.9). This corresponded to an adjusted mean difference of 2.79 (95% CI: 1.44–4.14; *p* < 0.001), equivalent to the consumption of nearly three additional food items. Total DDS also increased in the Nourish group, from 19.5 to 21.1, compared with a smaller increase in the No Nourish group (18.3 to 18.6; adjusted mean difference = 1.55, 95% CI: 0.65–2.44; *p* = 0.001). No significant changes in DDS were observed among 6–7-year-old children who received the Nourish intervention. The Engage intervention did not result in significant changes in DDS in either age group.

**Table 3:** Impact of the nourish and engage interventions on the diet diversity score and diet quality scores of 6–7 and 10–11 year old children

	**Diet Diversity Score**		**Nourish**	**No Nourish**	**Engage**	**No Engage**
**6-7 years **	Total	N	219	202	234	187
		Baseline mean (SD)	14.2 (3.6)	14.2 (3.8)	14.3 (3.4)	14.1 (3.3)
		Follow-up mean (SD)	14.2 (3.7)	14.5 (4.0)	14.1 (3.6)	14.7 (4.0)
		Adjusted difference in mean (95% CI)	- 0.41 (-1.42 – 0.59)	Reference	-0.54 (-1.54 – 0.46)	Reference
		*P* value	0.42		0.29	
**10-11 years**	Home	N	212	178	193	197
		Baseline mean (SD)	23.3 (6.1)	22.3 (6.0)	22.3 (5.7)	23.4 (6.3)
		Follow-up mean (SD)	24.7 (6.1)	23.4 (6.0)	24.0 (5.9)	24.3 (6.2)
		Adjusted difference in mean (95% CI)	0.31 (-0.74 – 1.37)	Reference	0.47 (-0.58 – 1.53)	Reference
		P-value	0.56		0.38	
	School	N	212	178	193	197
		Baseline mean (SD)	15.8 (7.2)	14.2 (6.4)	15.3 (6.7)	14.8 (7.0)
		Follow-up mean (SD)	17.5 (7.3)	13.9 (7.2)	16.0 (7.4)	15.6 (7.5)
		Adjusted difference in mean (95% CI)	2.79 (1.44 – 4.14)	Reference	0.14 (-1.21 – 1.49)	Reference
		*P*-value	<0.001		0.84	
	Total	N	212	178	193	197
		Baseline mean (SD)	19.5 (5.6)	18.3 (5.3)	18.8 (5.3)	19.1 (5.7)
		Follow-up mean (SD)	21.1 (5.9)	18.6 (5.6)	20.0 (5.8)	20.0 (6.0)
		Adjusted difference in mean 95% CI	1.55 (0.65 – 2.44)	Reference	0.26 (-0.64 – 1.16)	Reference
		*P*-value	0.001		0.57	
**6-7 years **	Total	N	219	202	234	187
		Baseline mean (SD)	3.2 (3.6)	3.4 (3.8)	3.4 (3.7)	3.2 (3.7)
		Follow-up mean (SD)	2.6 (3.5)	3.1 (2.5)	2.7 (3.5)	2.4 (3.2)
		Adjusted difference in mean (95% CI)	0.11 (-0.50 – 0.72)	Reference	0.26 (-0.36 – 0.87)	Reference
		P value	0.73		0.42	
**10-11 years**	Home	N	212	178	193	197
		Baseline mean (SD)	8.3 (6.3)	6.3 (5.1)	7.5 (6.2)	7.3 (5.4)
		Follow-up mean (SD)	8.9 (6.5)	7.2 (5.7)	8.2 (6.6)	8.0 (5.8)
		Adjusted diff. in mean (95% CI)	0.04 (-0.84 – 0.92)	Reference	0.03 (-0.84 – 0.91)	Reference
		*P*-value	0.92		0.94	
	School	N	212	178	193	197
		Baseline mean (SD)	10.6 (6.5)	9.0 (6.1)	10.0 (6.4)	9.8 (6.3)
		Follow-up mean (SD)	10.1 (6.0)	9.8 (6.3)	10.2 (6.4)	9.8 (5.8)
		Adjusted difference in mean (95% CI)	-0.58 (-2.09-0.92)	Reference	0.88 (-0.63 – 2.38)	Reference
		*P*-value	0.45		0.25	
	Total	N (baseline and follow-up responses)	212	178	193	197
		Baseline mean (SD)	9.5 (5.7)	7.6 (4.7)	8.7 (5.6)	8.6 (5.1)
		Follow-up mean (SD)	9.5 (5.5)	8.5 (5.2)	9.2 (5.8)	8.9 (4.9)
		Adjusted difference in mean 95% CI	-0.37 (-1.53 – 0.79)	Reference	0.53 (-0.62 – 1.68)	Reference
		*P*-value	0.54		0.37	

*Abbreviations*: *N *Number of completed responses (i.e. baseline and follow-up), *SD *Standard Deviation. In factorial analysis, the main effects of Nourish (compared to No Nourish) and Engage (compared to No Engage) are estimated. Models are adjusted for clustering at the school level and baseline scores. *P* value <0.05 indicative of significance

Subgroup analyses suggested that the increase in school DDS among 10–11-year-olds receiving Nourish was apparent in both boys and girls (Boys: adjusted mean difference = 2.48, 95% CI: 0.38–4.57, *p* = 0.02; Girls: adjusted mean difference = 3.08, 95% CI: 1.37–4.80, *p* < 0.001). For total DDS, increases remained statistically significant only among girls (adjusted mean difference = 1.89, 95% CI: 0.73–3.04, *p* = 0.001) (**Additional File 5**). The corresponding interaction test, however, did not reveal any statistically significant differences in total DDS (*p* = 0.59), suggesting that the Nourish intervention had similar effects on boys and girls. No gender-based differences were observed in the 6–7-year-old group.

No significant changes in DQS were detected in either 6–7-year or 10–11-year-old children who received the Nourish or Engage interventions compared to those who did not. Subgroup analysis likewise revealed no differences in DQS between boys and girls across both age groups for either intervention.

## Discussion

This secondary analysis of Project Daire suggests that the Nourish intervention was associated with favourable changes in dietary diversity among 10–11-year-old children, as indicated by increases in both school and total DDS. These findings align with the primary outcomes of the trial, which reported increased consumption of apples, mushrooms, white and brown breads, milk, chicken, and bacon/ham at school in this age group who received Nourish [15]. However, increased consumption of chocolate and fizzy drinks was also observed [15], which may explain why no measurable improvement in DQS was observed in the present analysis. The DQS applied negative scoring to foods recommended for limited intake, such as white bread and processed meats, which were not penalised under the DDS scoring system (scored as + 1 and 0, respectively). As a result, gains in dietary diversity may have been offset by concurrent increases in less nutritious foods, leading to no improvement in overall diet quality. Furthermore, no significant change in home DDS was observed among 10–11-year-olds, indicating that the observed increases in total DDS were largely driven by dietary changes within the school environment, with little or no extension into home eating behaviours. Among 6–7-year-old children in the Nourish group, no changes in DDS were observed. Similarly, no significant changes in DDS or DQS were detected in either age group in the Engage group.

Similar findings have been reported in the Food Dudes programme, a school-based behaviour change intervention conducted in the UK [28]. Children aged 4–11 years who participated in this programme not only increased their intake of FV but also showed an increase in the consumption of high-fat and high-sugar foods during lunchtime. However, the study did not assess whether these changes in dietary intake influenced the children’s overall dietary patterns, limiting the ability to make direct comparisons with our findings on diet diversity and quality. Most school-based nutrition research examining diet diversity or quality has been conducted in regions outside the UK and Europe, particularly in middle- and low-income countries. For example, a study in Ethiopia similarly reported increased diet diversity but limited impact on junk food consumption among girls aged 10–14 years following a school-based nutrition education [29]. However, the educational approach used in the study, along with differing contextual factors, such as target populations and outcome measures, complicates direct comparisons with our findings.

The whole-school food approach implemented in the Nourish intervention, including modifications to the school food environment and increased exposure to local food products through provision and food-related activities, likely contributed to the observed improvements in dietary diversity among older children. These findings are consistent with systematic reviews of school-based nutrition interventions, which suggest that multi-component approaches are more likely to yield positive changes in dietary outcomes [8, 10]. However, our results also suggest that greater dietary diversity does not necessarily translate to improved diet quality. This outcome may reflect the lack of restrictions within the Nourish intervention regarding the overall range of foods available or free choice. The rationale behind this strategy is rooted in Project Daire’s collaboration with stakeholders, including schools and the local food industry, with the aim of introducing children to Northern Ireland-sourced foods and fostering an understanding of food origins, rather than explicitly targeting reductions in less healthy food consumption. The focus on local food sourcing may also support more sustainable dietary behaviours by reducing the carbon footprint associated with food transport [30]. Future iterations of Daire should consider integrating additional strategies, with adherence to food and beverage standards, to encourage children to choose more nutritious foods in place of less healthy options. However, caution is warranted, as evidence suggests that restrictive approaches can be counterproductive, potentially increasing children’s desire for restricted items [28]. A whole-school approach that combines education with supportive environmental changes may help mitigate this risk by cultivating positive attitudes and reinforcing healthy behaviours [28, 31].

The absence of an increase in home DDS among 10–11-year-old children suggests that the positive effects of the Nourish intervention on school DDS did not extend into the home environment. Research indicates that school-based interventions that actively engage parents are more likely to be successful [10]. Parents can have an important influence over their children’s diets and eating habits, particularly among younger children, whose behaviours are more readily shaped by parental guidance [32]. Incorporating parental involvement in future iterations of Daire could help foster a supportive home environment that reinforces the positive behaviours learned at school, such as more diverse and nutritious dietary choices, thereby extending these habits into the home and potentially enhancing the overall impact of the intervention, particularly among younger children.

To our knowledge, Project Daire is the first initiative in the UK to evaluate environmental and educational strategies in primary schools through collaboration with public, private, and school stakeholders. Process evaluation indicated that the interventions were well-received, with no school clusters lost to follow-up [33]. Contamination of control participants was considered unlikely, supported by design features such as whole-school randomisation (rather than by class), which reduced risk of cross-group exposure, and independent data collection by trained researchers not involved in intervention delivery. Together, these measures reduced the likelihood that contamination influenced the outcomes [33]. Despite these strengths, logistical constraints, particularly school timetabling, delayed the start of delivery and consequently shortened the intended six-month intervention period to between 2.5 and 5 months. Evidence suggests that changes in children’s dietary behaviours are gradual, and shorter interventions may struggle to achieve meaningful effects. Longer interventions, often exceeding six months [10] or lasting a year or more [34, 35], are more likely to support meaningful behavioural changes. The shortened delivery period in this study may therefore have constrained its potential to generate significant effects [10], particularly in diet quality or among younger children. Nonetheless, some reviews, such as that by Calvert et al. (2019), caution that longer duration does not necessarily guarantee effectiveness, as intervention uptake, engagement, and exposure are also critical considerations [36]. From a statistical perspective, the present analyses employed multi-level models with random intercepts to account for clustering at the school level and were adjusted for baseline dietary scores. Nonetheless, residual confounding cannot be excluded, as factors such as individual-level socioeconomic status and detailed baseline dietary behaviours were not included. The trial was designed and powered only for the primary outcomes at the whole-sample level and not for the dietary outcomes presented here. Given the number of outcomes examined and the lack of adjustment for multiple comparisons, there is an increased risk of Type I error. These dietary analyses should therefore be regarded as exploratory and interpreted with caution. Future research should employ longer interventions with larger samples, while incorporating strategies to minimise residual confounding and appropriate statistical approaches to account for multiple testing, to strengthen the robustness of findings.

The study used food lists to record the foods ‘ever’ consumed by children. However, this binary scoring approach did not account for portion sizes or the frequency of consumption, which may have influenced the DQS results, as even small or occasional intakes of discretionary foods were penalised equally. This may have limited the ability to comprehensively assess the children’s overall dietary intake and, consequently, the impact of the interventions. Additionally, the food lists used differed between age groups in both length and complexity, which may have influenced the quality and detail of the dietary data collected. The simplified list used for younger children, designed to align with their cognitive and literacy levels, included fewer food items and may have constrained the ability to capture dietary diversity. In contrast, the more detailed, setting-specific lists used for older children, who typically have greater autonomy over their food choices across different environments, enabled a broader and more comprehensive assessment of intake. Younger children may also struggle with dietary recall or be unfamiliar with food names and preparation methods, as their meals are often managed by caregivers, such as parents, guardians, or teachers [21, 37]. While parental or guardian assistance was recommended with completing the food lists, it was not mandatory, potentially introducing variability in the level of support provided and inconsistency in the quality of reporting. Although efforts were made to minimise bias, including age-appropriate tools, providing standardised instructions, and blinding the data analyst, response bias (e.g., social desirability) remains a potential limitation. These factors may have contributed to under or over-reporting, particularly for discretionary items, and may partly explain the lack of observed changes in DDS among the younger age group compared to older children. Future research should employ more rigorous tools, such as FFQs, food diaries, or weighed food records, adapted to the cognitive and developmental capabilities of children. Proxy reporting may also be appropriate, particularly for younger age groups. Technology-assisted methods, such as mobile applications or online platforms, could help reduce the administrative burden on schools and researchers, minimize disruptions during data collection, and provide a more accurate representation of habitual dietary patterns [9, 38]. Finally, while the food lists were adapted from CADET, which has been validated in children from diverse social and ethnic backgrounds in England [21], future adaptations could explore to better reflect the cultural diversity of schoolchildren in Northern Ireland to ensure that a broader range of dietary practices and food items are captured.

To our knowledge, no dietary guideline index based on government recommendations has been specifically designed for school-aged children in the UK. Consequently, our scoring protocol was adapted from a secondary analysis of UK-based adult observational studies, which assessed adherence to the Eatwell Guide recommendations and their associations with health and environmental outcomes [22]. While aligning the scoring system with the Eatwell Guide ensures regional relevance and enhances the applicability of our findings within the UK context, the absence of validation for use in children remains a limitation. Despite these challenges, the DDS and DQS served complementary purposes in our study. The DDS was designed to capture positive shifts in dietary diversity across core food groups, even when discretionary foods were consumed. This measure may be particularly relevant in areas of socioeconomic disadvantage, such as those targeted by Project Daire, where improving access to a broader range of food groups may represent a meaningful step toward better nutrition. In such contexts, dietary diversity has been associated with indicators of welfare, diet quality, and nutrient adequacy [11, 39–41]. For example, the inclusion of foods like pancakes or milky puddings can increase dietary diversity relative to missed meals or highly monotonous diets. In this way, the DDS may serve as a practical screener for identifying basic dietary improvements, particularly in low-resource settings. In contrast, the DQS provided a stricter assessment of diet quality, emphasizing reductions in less healthy options alongside increases in nutrient-dense foods. Improvements in diet quality were only reflected in the DQS when increases in healthier food consumption outweighed the intake of discretionary items. The finding that DDS improved among older children, while DQS did not, suggests that promoting dietary diversity alone may be insufficient to improve overall diet quality if unhealthy foods remain prevalent. This distinction is important for informing the design of more effective school-based interventions. Future research should prioritise the development and validation of child-specific diet diversity and quality tools, to support more accurate and meaningful evaluations of dietary change in the context of school-based interventions.

## Conclusions

The findings of this study indicate that the Nourish intervention may help promote greater dietary diversity among older children through a whole-school food environment approach. However, the lack of improvement in diet quality suggests that increased diversity alone may not be sufficient to improve overall nutritional outcomes or alignment with dietary recommendations. Future iterations of Daire should aim to balance increased dietary diversity with reductions in discretionary food consumption. Tools such as the DDS can help monitor shifts in dietary diversity, particularly in disadvantaged settings, while supporting stakeholders’ goals to provide appealing food options with good uptake. Meanwhile, the DQS offers a complementary perspective by assessing overall dietary balance and identifying areas where less healthy food consumption remains high. To maximize intervention effectiveness, future efforts should prioritise validated dietary assessment tools, longer implementation periods, and additional strategies that target the consumption of less healthy foods and promote active parental engagement.

## Supplementary Information

Additional file 1. CONSORT Checklist for Cluster Randomised Controlled Trial

Additional file 2. TIDieR Checklist

Additional file 3. Nourish and Engage Interventions Components

Additional file 4 Questionnaires

Additional file 5. Gender Analyses

## Data Availability

The datasets used and/or analysed during the current study are available from the corresponding author on reasonable request.

## Abbreviations

UK: United Kingdom
FV: Fruit and vegetables
DDS: Diet Diversity Score
DQS: Diet Quality Score
CADET: Child and Diet Evaluation Tool
HFSS: High in fat, sugar and salt
95% CIs: 95% confidence intervals
