# Supplementary material for: Primary school-based food environment intervention increases diet diversity: Project Daire, a cluster randomized controlled trial
Source: Int J Behav Nutr Phys Act. 2025 Nov 21;22:149. doi: 10.1186/s12966-025-01842-4 (PMC12639729; doi:10.1186/s12966-025-01842-4)
Supplement: Supplementary file 3 — Additional file 3. Nourish and Engage Interventions Components [file 12966_2025_1842_MOESM3_ESM.docx]

**Additional file 1.** Nourish and Engage Interventions Components

Nourish intervention components

| Component | Overview |
| --- | --- |
| Healthy snack provision | Children received fruit (rotation of grapes and apples, 80g portion), milk or bread (rotation of wholegrain; high fibre; brown and white; wheaten with butter for spreading) packs twice a week during the school day. The snacks selected were provided by food industry partners and adhered to the Northern Ireland Food in Schools Policy (2013). |
| Enhancement of the school dining area | Schools were provided with tablecloths, centre pieces, bunting and menu boards for their canteens to enhance the school dining experience and create a café-style atmosphere. Printed posters were also provided to encourage children to make healthy food choices and explore where food comes from. |
| Enhancement of food presentation | School catering staff were provided with a pack containing serveware such as platters, tiered stands, reusable cups and paper straws; salad trolley and cutting equipment such as vegetable slicers and creative utensils such as coloured straws and skewers with the aim to encourage creative presentation of fruit, vegetables and other foods and their consumption in the school environment. |
| Food tasting day | Children had the opportunity to attend an interactive ‘Tasting Day’ event held at local Higher Education institutions. Food industry partners demonstrated their produce, encouraged children to taste new foods and participate in hands-on food preparation activities. Children were given a ‘food passport’ to record the new foods they tasted and a ‘goody bag’ containing local food products. |
| School cookery activities | Schools were provided with a recipe book developed by the research team and cookery equipment, such as mixing bowls, scales, graters, wooden spoons, rolling pins, to use in the cookery activities. Children were provided with take-home printed recipes and encouraged to try them at home with their parents/guardians. The recipes aimed to promote a balanced diet and interest in food preparation and utilised local foods, taken from the Council for the Curriculum, Examinations and Assessment (CCEA) ‘Growing for the Future’ resource. <http://ccea.org.uk/growing>. |
| Sensory Education | Schools were provided with a sensory education resource adapted from the Flavour School programme produced by Flavour School UK ([www.flavourschool.org.uk](http://www.flavourschool.org.uk)). The resource outlined five enjoyable and simple activities to help children learn about the senses, taste and flavour, and to encourage them to try new foods. |
| Food provision for school food event | Food industry partners provided food at school food themed event. |
| School food policies recommendation | School senior management received a document containing recommendations for changes that could be made to implement their school food policies. These recommendations covered areas such as food provision, dining environment, food-related events and reinforcing food policy messages to teachers and family. |

(Planned NOURISH components not delivered = Chef demonstration of healthy/local food recipe, Themed food days and nutritional analysis of food sampling)

Engage intervention topics and intended content overview

| **Lesson Topics** | **Intended content overview** |
| --- | --- |
| **Food Futures** (30-minute lessons but can be combined into a 1-hour lesson) | |
| **Favourite Food** (Lesson 1) | Children watch a video clip of a nutritionist who talks about her job, demonstrates the Eatwell Guide Plate (EWGP) visual aid and explains how foods can be placed into food groups. Children are asked to draw or write down all the foods and drinks they like and place them on a blank EWGP template provided. |
| **Food Ideas** (Lesson 2) | Children watch a video clip of a chef who talks about his job and favourite meal, reminds about the EWGP and prepares his favourite meal. Children are asked to think of a food and plan how they could use food to make their meal considering ‘who, what, why’ sheet (i.e. Who is the food for?’; What is it and what ingredients will be used; and Why would someone want to eat my food?). |
| **In the Restaurant** (Lesson 3) | Children watch a video clip of a restaurant manager who talks about her job. The children are asked to vote for the meal they would choose (from Lesson 2) for the menu in their restaurant giving reasons "who, what and why". |
| **Building Ideas** (Lesson 4) | This lesson revisits the meal winner(s) selected in Lesson 3. Children receive a copy of the winning idea(s) ‘who, what, why’ sheet and note any suggestions on it. They are encouraged to think about where the food comes from and how it could be healthy and tasty. |
| **Marketing** (Lesson 5) | Children watch a video clip of a marketer who discusses his job and are asked to outline the main roles of a marketer and what is involved in the role. They are asked to pretend that they are marketers and think about the meal choice they have chosen to market considering: Who is it for?, Why would people want/need it?’, Where will it be sold? and How will people know about it?. |
| **Advertising** (Lesson 6) | This lesson helps children to understand that advertising is a big part of marketing. Children are presented with logos or advertisement pictures to assess if they can recognize them and what captures their attentions. The lesson involves planning and designing children’s own advertisement for a meal product considering ‘Where?’, ‘What advertisements can we think of?’ and ‘Why we know/like these advertisements/logos. |
| **Great Teams** (Lesson 7) | Children gain insight into the people who work together to enable food to get from the farm or sea to their plates. They are divided into groups and work together to build towers with marshmallows and lollypops and to determine their teamwork efficiency. They watch video clips of the production processes of items like bread, mushrooms and salmon, and discusses about the jobs observed in the videos. |
| **Business Planning** (Lesson 8) | Children watch videos of different people who work in the food industry. They draw or write all the different jobs they can think of for their winning product. They are encouraged on how they could showcase their ideas developed throughout this topic with others in the school. |
| **Farm to Fork** (1-hour lessons) | |
| **Animal Welfare** | Children watch videos showcasing different types of farms and share their thoughts on the farms. The lesson aims to develop children’s awareness about different animals and their various roles in the food chain and animal welfare. |
| **Johnny Loves Milk** | Children learn about how milk is produced and read a book named 'Johnny Loves Milk.' In groups, they are asked to cut and stick their own milk production pictures. The children then watch 'Thompson’s Video' which features a story of animal feed production and are asked to identify the steps from the video and put them in order. |
| **Food Scribblers** | This lesson extends from the Story of Johnny Loves Milk and children look at the Sausage story and the Meat and Cheese Production Videos and play the Moy Park game and they are asked how many steps in the food chain they could spot. Children learn to express a story of one food chain in their own creative manner. |
| **Food Stories** | Children watch video clips of people who talk about their special traditional local food products or recipes. This allows children to relate to or notice differences between themselves and the individuals in the videos. Children will then research recipes from families or friends, or if they have a particular favourite local food as an example. Children will then present their findings and discuss their favourite food with a peer. |
| **Milk and farming** | This lesson is delivered by a visiting food scientist and covers the importance of animal feeding and its transfer to milk, the use of high-tech milk parlours to ensure the quality and safety of milk, various production methods for different dairy products (e.g., pasteurization, cheese making), and the health benefits of dairy products for bones and teeth. |
| **Where does food come from** | This lesson is delivered by a visiting food scientist and covers different food journeys (e.g., cheese), how animals or grains produce various food products (e.g., pasta from wheat), and includes an interactive class activity identifying different food items and their sources (e.g., bacon to pigs). |
| **Pleasure on a Plate** (1-hour lessons) | |
| **Sensory Scientist** | The lesson introduces children to a different career – a professional Sensory Scientist. Children become their own Sensory Scientist at home and use their creativity to describe everyday food. Additionally, children can ask their family members to describe the same food and discover that people taste food differently. In the next lesson, children compare the similarities and differences in how they and other people described their food. |
| **Portion Size** | Children learn about the portion size of foods, which will help them to determine the right amount of food they should consume. They also learn about volume and capacity, the concept of energy consumption, and that some foods should be consumed occasionally. |
| **Seasonality** | The lesson aims to develop children’s understanding of importing and exporting food. Children create a seasonality clock mosaic, and in groups, they explore different fruits and vegetables consumed in respective seasons. Afterwards, the class can come together to paint or assemble the clock. |
| **Growing** | Children watch a video and learn about seed growing, the different parts of a plant and what is required for the plant to grow.  Children also gain insights into the work required to grow food by planting seeds and caring for their growing fruit and vegetables through experiential learning. |
| **Portion size and labelling** | This lesson is delivered by a visiting psychologist and covered topics such as food labels, portion size, and healthy choices and marketing. During the session, children play games focused on recognizing food logos. |
| **Physical education and food** | The lesson, delivered by a guest researcher, includes interactive physical education games. Children learn about the importance of exercise, nutrition and hydration for exercise, and food supply chains. Children can also create a new game or adapt a common game to make it food-related for incorporation in physical education lessons. |
| **Visit Options** | |
| **Industry visits** | Schools are offered visits to a range of local food industry partners premises (e.g. bakery, mushroom farm, poultry producer and fishery). |
| **Mobile farm** | A mobile farm visits schools where children can interact with the animals. The farm staff provide information about the animals and on their care. |
