# Supplementary material for: Primary school-based food environment intervention increases diet diversity: Project Daire, a cluster randomized controlled trial
Source: Int J Behav Nutr Phys Act. 2025 Nov 21;22:149. doi: 10.1186/s12966-025-01842-4 (PMC12639729; doi:10.1186/s12966-025-01842-4)
Supplement: Supplementary file 4 — Additional file 4 Questionnaires [file 12966_2025_1842_MOESM4_ESM.docx]

**6-7 year old**

Please complete the list below by placing a tick ✓ for your answer in the right box.

The question is: **Do you ever eat or drink any of the foods/drinks on the list?**

Example:

| **Do you ever eat or drink any of the foods/drinks on this list?** | |  |  | **I’m not sure if I eat or drink this** |
| --- | --- | --- | --- | --- |
|  |  | **Yes!**  **I sometimes eat or drink this** | **No!**  **No I never eat or drink this** |  |
| Baked beans | 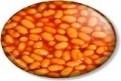 |  | ✓ |  |
| Biscuits | 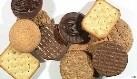 | ✓ |  |  |

**PLEASE START ON NEXT PAGE!**

| **Do you ever eat or drink any of the food/drinks on this list?** | | **Yes!**  **I sometimes eat or drink this** | **No!**  **I never eat or drink this** | **I’m not sure if I eat or drink this** |
| --- | --- | --- | --- | --- |
|  |  |  |  |  |
| 1. Fruit e.g. apples, grapes, bananas, strawberries and others | 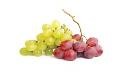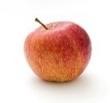  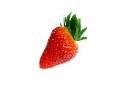  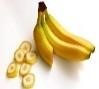 |  |  |  |
| 2. Vegetables e.g. broccoli, carrots, peas, sweetcorn, stir-fry, salad, baked beans,  others | 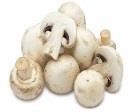 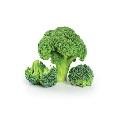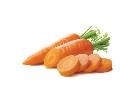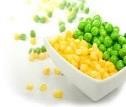     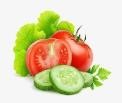 |  |  |  |

| **Do you ever eat or drink any of the food/drinks on this list?** | | **Yes!**  **I sometimes eat or drink this** | **No!**  **I never eat or drink this** | **I’m not sure if I eat or drink this** |
| --- | --- | --- | --- | --- |
| 3. Potatoes, mash, jacket potato, chips, waffles, potato faces | 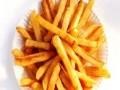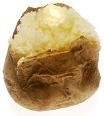 |  |  |  |
| 4. Rice/Pasta  (white/brown) | 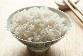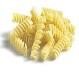 |  |  |  |
| 5. White/wholemeal bread | 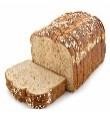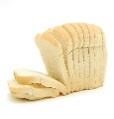 |  |  |  |
| 6. Porridge/Ready  Brek/Weetabix | 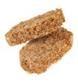  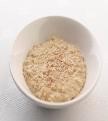 |  |  |  |
| 7. Other breakfast cereal | 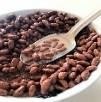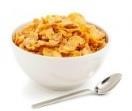 |  |  |  |

| **Do you ever eat or drink any of the food/drinks on this list?** | | **Yes!**  **I sometimes eat or drink this** | **No!**  **I never eat or drink this** | **I’m not sure if I eat or drink this** |
| --- | --- | --- | --- | --- |
| 8. Pancakes/scones/ fruit bread | 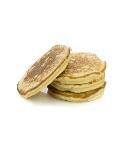 |  |  |  |
| 9. Milk to drink/on cereal | 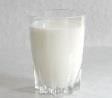  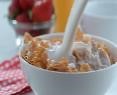 |  |  |  |
| 10. Cheese | 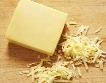 |  |  |  |
| 11. Yoghurts | 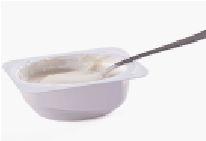 |  |  |  |
| 12. Ice cream/milky pudding e.g. custard. | 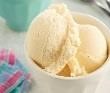 |  |  |  |
| 13. Chicken sliced (no sauce) | 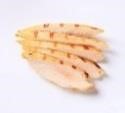 |  |  |  |
| 14. Chicken nuggets/burgers | 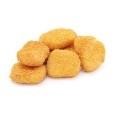 |  |  |  |
| 15. Beef (sliced, minced, chops, stew/casserole/c urry) | 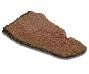  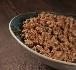 |  |  |  |

| **Do you ever eat or drink any of the food/drinks on this list?** | | **Yes!**  **I sometimes eat or drink this** | **No!**  **I never eat or drink this** | **I’m not sure if I eat or drink this** |
| --- | --- | --- | --- | --- |
| 16. Bacon/ham/ sausages | 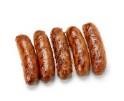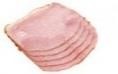 |  |  |  |
| 17. Lamb | 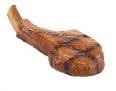 |  |  |  |
| 18. Eggs (scrambled, poached, boiled, fried) | 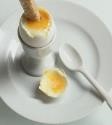 |  |  |  |
| 19. Fish fillet/tuna (salmon, cod, haddock) | 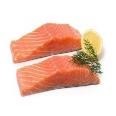 |  |  |  |
| 20. Fishfingers/fish in batter | 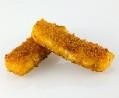 |  |  |  |
| 21. Crisps | 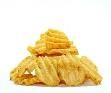 |  |  |  |
| 22. Biscuits/ chocolate | 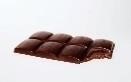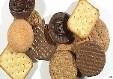 |  |  |  |
| 23. Sweets | 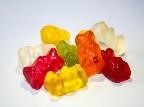 |  |  |  |
| 24. Nuts | 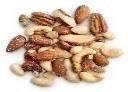 |  |  |  |
| **Do you ever eat or drink any of the food/drinks on this list?** | | **Yes!**  **I sometimes eat or drink this** | **No!**  **I never eat or drink this** | **I’m not sure if I eat or drink this** |
| 25. Cakes/buns/muffins | 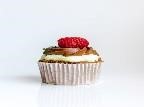 |  |  |  |
| 26. Water | 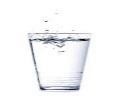 |  |  |  |
| 27. Juice  (dilutant, pure) | 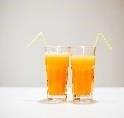 |  |  |  |
| 28. Fizzy drink | 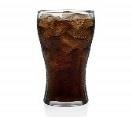 |  |  |  |
| 29. Milkshakes | 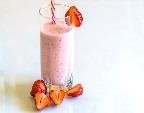 |  |  |  |

And finally……

Do you usually eat breakfast every morning?

| \| No \|  \| Yes \| \| --- \| --- \| --- \| | **THE END**    **Thanks for your help!**    ☺ |
| --- | --- | --- | --- | --- |

**10-11 year old**

Please complete the list below by placing a tick ✓ for your answer in the right box.

The question is: **Do you ever eat or drink any of the foods/drinks on the list AT HOME?**

Example:


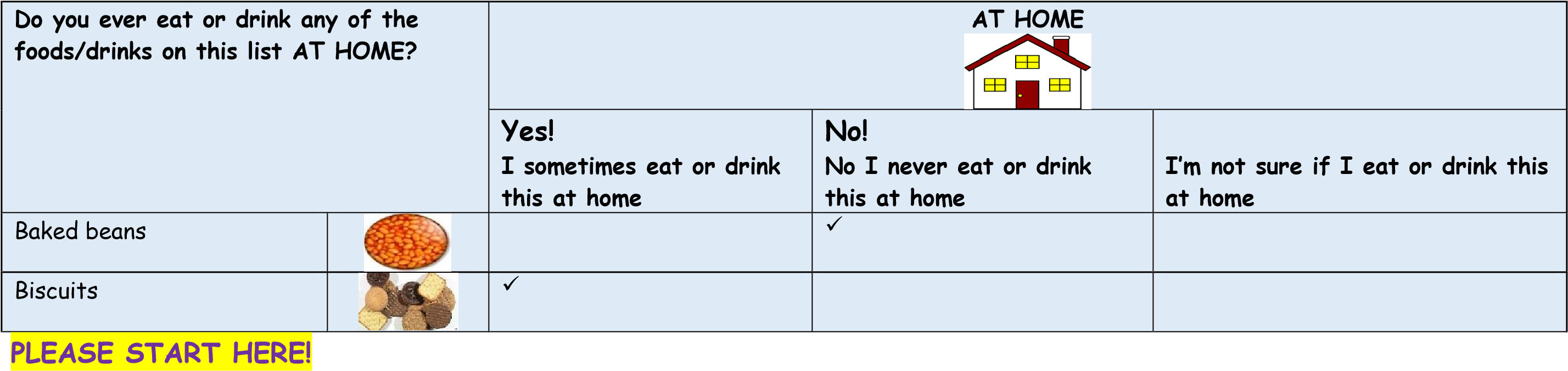


| **Do you ever eat or drink any of the foods/drinks on this list AT HOME?** | |  | **AT HOME**  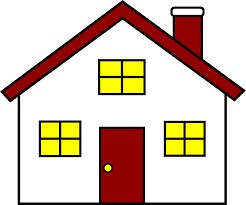 |  |
| --- | --- | --- | --- | --- |
|  |  | **Yes!**  **I sometimes eat or drink this at home** | **No!**  **No I never eat or drink this at home** | **I’m not sure if I eat or drink this at home** |
| **FRUIT** | |  |  |  |
| 1. Apples | 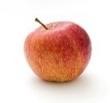 |  |  |  |

| **Do you ever eat or drink any of the foods/drinks on this list AT HOME?**  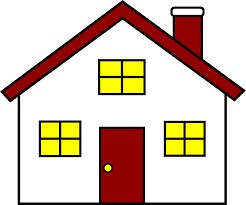 | | **Yes!**  **I sometimes eat or drink this at home** | **No!**  **No I never eat or drink this at home** | **I’m not sure if I eat or drink this at home** |
| --- | --- | --- | --- | --- |
| 2. Grapes | 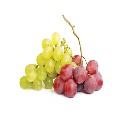 |  |  |  |
| 3. Bananas | 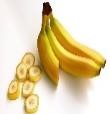 |  |  |  |
| 4. Strawberries | 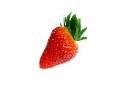 |  |  |  |
| 5. Pineapple | 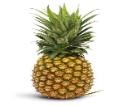 |  |  |  |
| **VEGETABLES** | | | | |
| 6. Mushrooms | 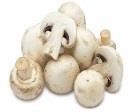 |  |  |  |
| 7. Broccoli | 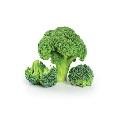 |  |  |  |
| 8. Carrots | 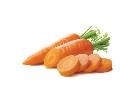 |  |  |  |
| 9. Peas/sweetcorn | 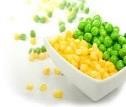 |  |  |  |
| 10. Stir-fried vegetables | 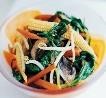 |  |  |  |

| **Do you ever eat or drink any of the foods/drinks on this list AT HOME?**  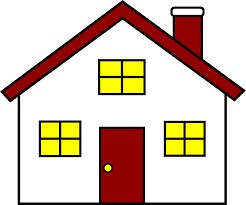 | | **Yes!**  **I sometimes eat or drink this at home** | **No!**  **No I never eat or drink this at home** | **I’m not sure if I eat or drink this at home** |
| --- | --- | --- | --- | --- |
| 11. Salad (tomatoes, leaves OR cucumber) | 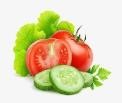 |  |  |  |
| 12. Baked beans | 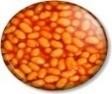 |  |  |  |
| **STARCHY CARBOHYDRATES** | | | | |
| 13. Potatoes | 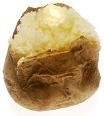 |  |  |  |
| 14. Chips/Potato Faces/Potato  Waffles | 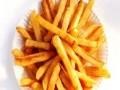 |  |  |  |
| 15. Rice/Pasta  (white/brown) | 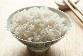  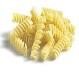 |  |  |  |
| 16. White bread | 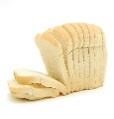 |  |  |  |
| 17. Brown/wholemeal bread | 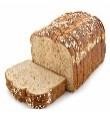 |  |  |  |
| 18. Porridge/Ready Brek | 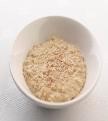 |  |  |  |
| 19. Cornflakes/Rice Krispies | 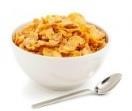 |  |  |  |

| **Do you ever eat or drink any of the foods/drinks on this list AT HOME?**  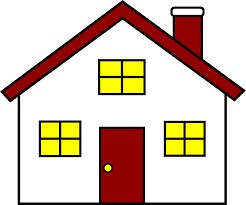 | | **Yes!**  **I sometimes eat or drink this at home** | **No!**  **No I never eat or drink this at home** | **I’m not sure if I eat or drink this at home** |
| --- | --- | --- | --- | --- |
| 20. Frosties/Cocopops | 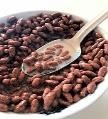 |  |  |  |
| 21. Weetabix/Bran Flakes | 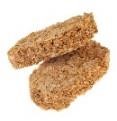 |  |  |  |
| 22. Pancakes/scones/ fruit bread | 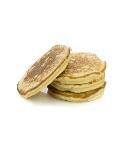 |  |  |  |
| 23. Crackers/breadst  icks | 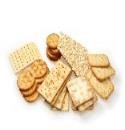 |  |  |  |
| **DAIRY** |  |  |  |  |
| 24. Milk to drink | 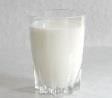 |  |  |  |
| 25. Milk on cereal | 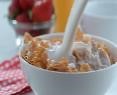 |  |  |  |
| 26. Cheddar cheese | 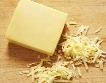 |  |  |  |
| 27. Cheese spread | 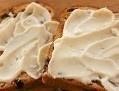 |  |  |  |
| 28. Margarine/butter on  toast/sandwiches | 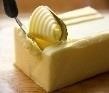 |  |  |  |
| 29. Yoghurts | 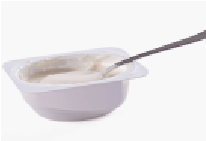 |  |  |  |

| **Do you ever eat or drink any of the foods/drinks on this list AT HOME?**  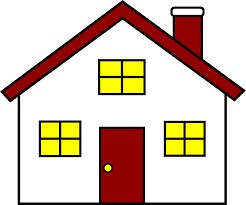 | | **Yes!**  **I sometimes eat or drink this at home** | **No!**  **No I never eat or drink this at home** | **I’m not sure if I eat or drink this at home** |
| --- | --- | --- | --- | --- |
| 30. Ice cream/milky pudding e.g. custard. | 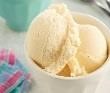 |  |  |  |
| **MEAT/FISH/EGGS/**  **PULSES** |  |  |  |  |
| 31. Chicken sliced (no sauce) | 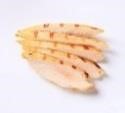 |  |  |  |
| 32. Chicken nuggets/burgers | 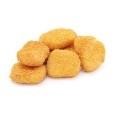 |  |  |  |
| 33. Beef (sliced, minced, chops, stew/casserole/c urry) | 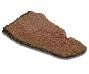  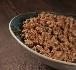 |  |  |  |
| 34. Bacon/ham | 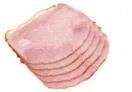 |  |  |  |
| 35. Sausages | 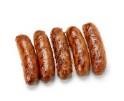 |  |  |  |
| 36. Lamb | 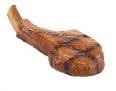 |  |  |  |
| 37. Meat in pastry (e.g. sausage rolls, meat pie) | 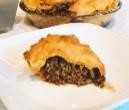 |  |  |  |
| 38. Eggs (scrambled, poached, boiled, fried) | 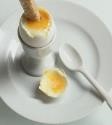 |  |  |  |

| **Do you ever eat or drink any of the foods/drinks on this list AT HOME?**  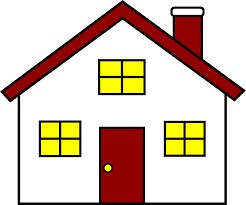 | | **Yes!**  **I sometimes eat or drink this at home** | **No!**  **No I never eat or drink this at home** | **I’m not sure if I eat or drink this at home** | |
| --- | --- | --- | --- | --- | --- |
| 39. Fish fillet (salmon, cod, haddock) | 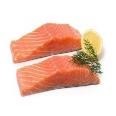 |  |  |  | |
| 40. Tuna | 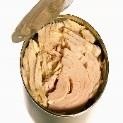 |  |  |  | |
| 41. Fishfingers/fish in batter | 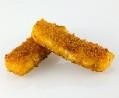 |  |  |  | |
| **SNACKS** |  |  |  |  | |
| 42. Crisps | 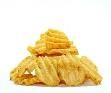 |  |  |  | |
| 43. Biscuits (with/without chocolate) | 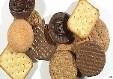 |  |  |  | |
| 44. Sweets (jelly/gums/ toffees) | 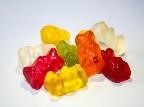 |  |  |  | |
| 45. Nuts |  |  |  |  | |
| 46. Chocolate |  |  |  |  | |
| 47. Cakes/buns/muffins |  |  |  |  | |
| **DRINKS** |  |  |  |  | |
| 48. Water |  |  |  |  | |
| **Do you ever eat or drink any of the foods/drinks on this list AT HOME?** | | **Yes!**  **I sometimes eat or drink this at home** | **No!**  **No I never eat or drink this at home** | **I’m not sure if I eat or drink this at home** | |
| 49. Juice (dilutant, pure) |  |  |  |  | |
| 50. Fizzy drink (Cola, Fanta, sugar and sugar free  varieties) |  |  |  |  | |
| 51. Tea/coffee |  |  |  |  | |
| 52. Milkshakes |  |  |  |  | |
| **PLEASE TURN OVER TO COMPLETE CHECKLIST FOR WHAT YOU EAT AT SCHOOL!** ☺ | | | | |  |

**The next question is: Do you ever eat or drink any of the foods/drinks on the list AT SCHOOL?**

| **Do you ever eat or drink any of the foods/drinks on this list AT SCHOOL?** | |  | **AT SCHOOL** |  |
| --- | --- | --- | --- | --- |
|  |  | **Yes!**  **I sometimes eat or drink this at school** | **No!**  **No I never eat or drink this at school** | **I’m not sure if I eat or drink this at school** |
| **FRUIT** | | |  |  |
| 1. Apples |  |  |  |  |
| 2. Grapes |  |  |  |  |
| 3. Bananas |  |  |  |  |
| 4. Strawberries |  |  |  |  |
| 5. Pineapple |  |  |  |  |
| **VEGETABLES** | | |  |  |
| 6. Mushrooms |  |  |  |  |
| 7. Broccoli |  |  |  |  |

| **Do you ever eat or drink any of the foods/drinks on this list AT SCHOOL?** | | **Yes!**  **I sometimes eat or drink this at school** | **No!**  **No I never eat or drink this at school** | **I’m not sure if I eat or drink this at school** |
| --- | --- | --- | --- | --- |
| 8. Carrots |  |  |  |  |
| 9. Peas/sweetcorn |  |  |  |  |
| 10. Stir-fried vegetables |  |  |  |  |
| 11. Salad (tomatoes, leaves OR cucumber) |  |  |  |  |
| 12. Baked beans |  |  |  |  |
| **STARCHY CARBOHYDRATES** | | | | |
| 13. Potatoes |  |  |  |  |
| 14. Chips/Potato Faces/Potato  Waffles |  |  |  |  |
| 15. Rice/Pasta  (white/brown) |  |  |  |  |
| 16. White bread |  |  |  |  |
| 17. Brown/wholemeal bread |  |  |  |  |

| **Do you ever eat or drink any of the foods/drinks on this list AT SCHOOL?** | | **Yes!**  **I sometimes eat or drink this at school** | **No!**  **No I never eat or drink this at school** | **I’m not sure if I eat or drink this at school** |
| --- | --- | --- | --- | --- |
| 18. Porridge/Ready Brek |  |  |  |  |
| 19. Cornflakes/Rice Krispies |  |  |  |  |
| 20. Frosties/Cocopops |  |  |  |  |
| 21. Weetabix/Bran Flakes |  |  |  |  |
| 22. Pancakes/scones/ fruit bread |  |  |  |  |
| 23. Crackers/ breadsticks |  |  |  |  |
| **DAIRY** |  |  |  |  |
| 24. Milk to drink |  |  |  |  |
| 25. Milk on cereal |  |  |  |  |
| 26. Cheddar cheese |  |  |  |  |

| **Do you ever eat or drink any of the foods/drinks on this list AT SCHOOL?** | | **Yes!**  **I sometimes eat or drink this at school** | **No!**  **No I never eat or drink this at school** | **I’m not sure if I eat or drink this at school** |
| --- | --- | --- | --- | --- |
| 27. Cheese spread |  |  |  |  |
| 28. Margarine/butter on  toast/sandwiches |  |  |  |  |
| 29. Yoghurts |  |  |  |  |
| 30. Ice cream/milky pudding |  |  |  |  |
| **MEAT/FISH/EGGS/**  **PULSES** |  |  |  |  |
| 31. Chicken sliced (no sauce) |  |  |  |  |
| 32. Chicken nuggets/burgers |  |  |  |  |
| 33. Beef (sliced, minced, chops, stew/casserole/ curry) |  |  |  |  |
| 34. Bacon/ham |  |  |  |  |
| 35. Sausages |  |  |  |  |

| **Do you ever eat or drink any of the foods/drinks on this list AT SCHOOL?** | | **Yes!**  **I sometimes eat or drink this at school** | **No!**  **No I never eat or drink this at school** | **I’m not sure if I eat or drink this at school** |
| --- | --- | --- | --- | --- |
| 36. Lamb |  |  |  |  |
| 37. Meat in pastry (e.g. sausage rolls, meat pie) |  |  |  |  |
| 38. Eggs (scrambled, poached, boiled, fried) |  |  |  |  |
| 39. Fish fillet (salmon, cod, haddock) |  |  |  |  |
| 40. Tuna |  |  |  |  |
| 41. Fishfingers/fish in batter |  |  |  |  |
| **SNACKS** |  |  |  |  |
| 42. Crisps |  |  |  |  |
| 43. Biscuits (with/without chocolate) |  |  |  |  |
| 44. Sweets  (jelly/gums/toffe es) |  |  |  |  |
| **Do you ever eat or drink any of the foods/drinks on this list AT SCHOOL?** | | **Yes!**  **I sometimes eat or drink this at school** | **No!**  **No I never eat or drink this at school** | **I’m not sure if I eat or drink this at school** |
| 45. Nuts |  |  |  |  |
| 46. Chocolate |  |  |  |  |
| 47. Cakes/buns/ Muffins |  |  |  |  |
| **DRINKS** |  |  |  |  |
| 48. Water |  |  |  |  |
| 49. Juice (dilutant, pure) |  |  |  |  |
| 50. Fizzy drink (Cola, Fanta, sugar and sugar free  varieties) |  |  |  |  |
| 51. Tea/coffee |  |  |  |  |
| 52. Milkshakes |  |  |  |  |

And finally……Please turn over the page for the last question!

Do you usually eat breakfast every morning?

|  | | |  |  |
| --- | --- | --- | --- | --- |
|  | No |  |  | Yes |

**THE END**

**Thanks for your help!**

☺
