# Supplementary material for: Primary school-based food environment intervention increases diet diversity: Project Daire, a cluster randomized controlled trial
Source: Int J Behav Nutr Phys Act. 2025 Nov 21;22:149. doi: 10.1186/s12966-025-01842-4 (PMC12639729; doi:10.1186/s12966-025-01842-4)
Supplement: Supplementary file 5 — Additional file 5. Gender Analyses [file 12966_2025_1842_MOESM5_ESM.docx]

**Additional File 5.** Impact of the Nourish and Engage Interventions on the Diet Diversity Score and Diet Quality Scores of 6-7 and 10-11 year old children by gender.

|  |  | | **Boys** | | | | **Girls** | | | |
| --- | --- | --- | --- | --- | --- | --- | --- | --- | --- | --- |
|  | **Diet Diversity Score** | | **Nourish** | **No Nourish** | **Engage** | **No Engage** | **Nourish** | **No Nourish** | **Engage** | **No Engage** |
| **6-7 years** | **Total** | **N**  **Baseline mean (SD)**  **Follow-up mean (SD)**  **Adjusted difference in mean (95% CI)** | 107  14.5 (3.3)  14.7 (3.7)  -0.09 (-1.25 – 1.06) | 102  13.9 (3.5)  14.4 (4.1)  Reference | 107  14.4 (3.4)  14.3 (4.1)  -0.58 (-1.73 – 0.57) | 102  14.0 (3.5)  14.7 (3.7)  Reference | 115  14.0 (3.4)  13.7 (3.8)  -0.55 (-1.65 – 0.55) | 97  14.6 (3.2)  14.7 (3.9)  Reference | 127  14.3 (3.4)  13.8 (4.0)  -0.67 (-1.77 – 0.43) | 85  14.3 (3.2)  14.7 (3.6)  Reference |
|  |  | **P value** | 0.88 | | 0.32 | | 0.33 | | 0.24 | |
| **10-11 years** | **Home** | **N**  **Baseline mean (SD)**  **Follow-up mean (SD)**  **Adjusted diff. in mean (95% CI)** | 84  23.1 (6.7)  24.0 (6.4)  0.15 (-1.22 – 1.52) | 82  21.9 (6.1)  23.3 (6.4)  Reference | 86  22.3 (5.7)  24.5 (6.2)  -0.78 (-0.59 – 2.15) | 80  22.8 (7.1)  23.3 (6.9)  Reference | 94  23.3 (5.5)  24.7 (5.3)  0.79 (-0.28 – 1.85) | 96  22.7 (5.7)  23.5 (5.6)  Reference | 107  21.9 (5.7)  23.5 (5.6)  0.37 (-0.73 – 1.47) | 83  24.3 (5.2)  24.8 (5.3)  Reference |
|  |  | **P-value** | 0.83 | | 0.26 | | 0.15 | | 0.52 | |
|  | **School** | **N**  **Baseline mean (SD)**  **Follow-up mean (SD)**  **Adjusted difference in mean (95% CI)** | 84  15.9 (7.9)  17.4 (8.3)  2.48 (0.38 – 4.57) | 82  14.0 (6.8)  13.8 (8.1)  Reference | 86  16.1 (7.5)  16.3 (8.5)  -0.45 (-2.56 – 1.66) | 80  13.8 (7.3)  14.9 (8.2)  Reference | 94  15.1 (6.0)  17.3 (6.4)  3.08 (1.37 – 4.80) | 96  14.3 (6.0)  13.9 (6.4)  Reference | 107  14.6 (6.0)  15.8 (6.4)  0.40 (-1.33 – 2.12) | 83  14.9 (6.0)  15.3 (6.9)  Reference |
|  |  | **P-value** | 0.02 | | 0.68 | | 0.000 | | 0.65 | |
|  | **Total** | **N (baseline and follow-up responses)**  **Baseline mean (SD)**  **Follow-up mean (SD)**  **Adjusted difference in mean 95% CI** | 84  19.5 (6.3)  21.0 (6.8)  1.15 (-0.23 – 2.54) | 82  18.0 (5.6)  18.6 (6.4)  Reference | 86  19.4 (5.6)  20.4 (6.7)  -0.03 (-1.41 – 1.35) | 80  18.0 (6.3)  19.1 (6.6)  Reference | 94  19.2 (4.6)  21.0 (4.9)  1.89 (0.73 – 3.04) | 96  18.5 (5.1)  18.7 (5.0)  Reference | 107  18.2 (5.0)  19.6 (5.1)  0.25 (-0.92 – 1.43) | 83  19.6 (4.6)  20.1 (5.0)  Reference |
|  |  | **P-value** | 0.10 | | 0.97 | | 0.001 | | 0.67 | |

|  |  | | **Boys** | | | | **Girls** | | | |
| --- | --- | --- | --- | --- | --- | --- | --- | --- | --- | --- |
|  | **Diet Quality Score** | | **Nourish** | **No Nourish** | **Engage** | **No Engage** | **Nourish** | **No Nourish** | **Engage** | **No Engage** |
| **6-7 years** | **Total** | **N**  **Baseline mean (SD)**  **Follow-up mean (SD)**  **Adjusted difference in mean (95% CI)** | 104  3.3 (3.4)  2.6 (3.2)  -0.16 (-1.03 – 0.71) | 105  3.3 (3.8)  2.8 (3.4)  Reference | 107  3.7 (3.6)  2.8 (3.2)  -0.03 (-0.91 – 0.85) | 102  2.9 (3.6)  2.6 (3.4)  Reference | 105  3.2 (3.8)  2.6 (3.8)  0.36 (-0.48 – 1.21) | 97  3.5 (3.9)  2.2 (2.8)  Reference | 115  3.2 (3.8)  2.6 (3.8)  0.66 (-0.20 – 1.52) | 97  3.5 (3.9)  2.2 (2.8)  Reference |
|  |  | **P value** | 0.72 | | 0.94 | | 0.40 | | 0.13 | |
| **10-11 years** | **Home** | **N**  **Baseline mean (SD)**  **Follow-up mean (SD)**  **Adjusted diff. in mean (95% CI)** | 84  7.7 (6.2)  8.1 (6.3)  -0.35 (-1.76– 1.06) | 82  5.1 (4.8)  6.6 (6.0)  Reference | 86  6.8 (6.4)  7.5 (6.5)  -0.23 (-1.60–1.15) | 80  6.0 (4.7)  7.2 (5.8) Reference | 94  8.7 (6.3)  9.5 (6.7)  0.44 (-0.67–1.55) | 96  7.2 (5.1)  7.8 (5.4)  Reference | 107  8.0 (6.0)  8.8 (6.6)  0.32 (-0.79-1.43) | 83  7.9 (5.4)  8.4 (5.4)  Reference |
|  |  | **P-value** | 0.62 | | 0.75 | | 0.44 | | 0.57 | |
|  | **School** | **N**  **Baseline mean (SD)**  **Follow-up mean (SD)**  **Adjusted difference in mean (95% CI)** | 84  10.6 (6.7)  9.9 (6.2)  -0.97 (-2.88-0.94) | 82  8.0 (5.9)  9.8 (7.0)  Reference | 86  9.5 (6.3)  10.0 (6.7)  0.37 (-1.50 – 2.25) | 80  9.2 (6.7)  9.7 (6.4)  Reference | 94  9.9 (6.3)  9.8 (5.5)  -0.17 (-1.90 – 1.55) | 96  9.9 (6.1)  9.9 (5.7)  Reference | 107  10.4 (6.6)  10.3 (6.2)  1.10 (-0.63-2.83) | 83  9.3 (5.6)  9.2 (4.7)  Reference |
|  |  | **P-value** | 0.32 | | 0.70 | | 0.84 | | 0.21 | |
|  | **Total** | **N (baseline and follow-up responses)**  **Baseline mean (SD)**  **Follow-up mean (SD)**  **Adjusted difference in mean 95% CI** | 84  9.1 (5.7)  9.0 (5.4)  -0.87 (-2.28-0.54) | 82  6.6 (4.4)  8.2 (5.7)  Reference | 86  8.1 (5.5)  8.8 (5.9)  0.11 (-1.26 - 1.49) | 80  7.6 (5.0)  8.5 (5.2)  Reference | 94  9.3 (5.6)  9.6 (5.5)  0.22 (-0.97-1.41) | 96  8.6 (4.7)  8.8 (4.7)  Reference | 107  9.2 (5.6)  9.6 (5.7)  0.59 (-0.60-1.78) | 83  8.6 (4.6)  8.8 (4.2)  Reference |
|  |  | **P-value** | 0.23 | | 0.87 | | 0.72 | | 0.33 | |

Abbreviations: N: Number of completed responses (i.e. baseline and follow-up responses); SD: Standard Deviation. In factorial analysis, the 2 main effects (Nourish compared with No Nourish and Engage compared with No Engage) are investigated. Adjusted for clustering and baseline values. *P* value <0.05 indicative of significanc
